# Effects of aquatic exercise therapy on lung function in patients with asthma: a systematic review and meta-analysis

**DOI:** 10.3389/fphys.2026.1931034

**Published:** 2026-08-28

**Authors:** Xiaofeng Cheng, Jinsong Luo, Xianhui Liao

**Affiliations:** 1 Department of Infectious Diseases, Shangyu People’s Hospital of Shaoxing, Shaoxing University, Shaoxing, Zhejiang, China; 2 Wuchang University of Technology, Wuhan, Hubei, China; 3 Department of Physiotherapy, School of Primary and Allied Health Care, Monash University, Melbourne, VIC, Australia

**Keywords:** aquatic exercise, asthma, meta-analysis, pulmonary function, pulmonary rehabilitation, systematic review

## Abstract

**Background:**

Exercise is recommended as an adjunctive therapy for asthma, yet the effects of aquatic exercise on pulmonary function remain uncertain because findings from randomized controlled trials (RCTs) have been inconsistent. This systematic review and meta-analysis aimed to synthesize the current evidence regarding the effects of aquatic exercise on pulmonary function in patients with asthma.

**Methods:**

This systematic review was conducted in accordance with the PRISMA 2020 statement. PubMed, Embase, Web of Science, Cochrane Library, and CINAHL were systematically searched from database inception to 4 July 2026. Randomized controlled trials comparing structured aquatic exercise with routine care, health education, or land-based exercise in patients with asthma were included. The primary outcomes were forced expiratory volume in one second (FEV_1_), FEV_1_ (% predicted). Secondary outcomes included FEV_1_/FVC (%), forced vital capacity (FVC), FVC (% predicted), peak expiratory flow (PEF), PEF (% predicted), and forced expiratory flow at 25–75% of FEF (FEF_2575_% predicted). Age-based subgroup analyses were performed for FEV_1_ and PEF.

**Results:**

Thirteen RCTs involving 391 participants, were included. Aquatic exercise significantly improved FEV_1_ (MD = 0.21 L, 95% CI 0.02–0.41), PEF (MD = 0.51, 95% CI 0.12–0.91), FVC (% predicted) (MD = 12.31%, 95% CI 7.25–17.37), and FEF_2575_% predicted (MD = 10.75%, 95% CI 3.05–18.45). No significant improvements were observed for FEV_1_ (% predicted), FEV_1_/FVC (%), FVC, or PEF (% predicted). Subgroup analyses demonstrated that the improvement in FEV_1_ was significant in participants aged <18 years but not in adults, whereas PEF improved in both age groups.

**Conclusions:**

Aquatic exercise improves several indicators of pulmonary function in patients with asthma, particularly absolute FEV_1_, PEF, FVC (% predicted), and FEF_2575_% predicted. Children and adolescents may derive greater benefits in FEV_1_ than adults, suggesting that aquatic exercise represents a promising adjunctive pulmonary rehabilitation strategy, especially for younger individuals with asthma.

## 1 Introduction

Asthma is a heterogeneous chronic inflammatory airway disease characterized by recurrent wheezing, shortness of breath, chest tightness, and cough, accompanied by variable airflow limitations (Lung and B. Institute, 1995). Despite significant advances in pharmacological treatment, asthma remains one of the most common chronic respiratory diseases globally, affecting over 260 million people and leading to increased healthcare costs, reduced physical activity, decreased quality of life, and preventable death (Lung and B. Institute, 1995; Soriano et al., 2020). Current international guidelines emphasize that the goals of asthma management extend beyond symptom control to include protecting lung function, preventing acute exacerbation, and maintaining normal physical activity (Reddel et al., 2022). Therefore, effective non-pharmacological interventions have become an increasingly important component of comprehensive asthma management (Carson et al., 1996).

Regular exercise is widely recognized as an effective adjunct to pharmacological therapy for asthma (Reddel et al., 2022). Growing evidence suggests that appropriately prescribed exercise training can improve cardiopulmonary function, respiratory muscle function, exercise capacity, asthma control, and health-related quality of life without increasing the risk of acute exacerbation (Carson et al., 1996; Feng et al., 2021). Recent systematic and umbrella reviews further indicate that structured exercise programs, including aerobic exercise, resistance training, breathing exercises, and yoga, may also improve certain lung function parameters, although the degree of benefit varies considerably among different exercise types and patient populations (Huang et al., 2025).

Among these interventions, aquatic exercise has garnered increasing clinical attention due to its numerous physiological characteristics, which may be particularly beneficial for asthma patients (Becker, 2009). Hydrostatic pressure can increase inspiratory muscle load and potentially enhance respiratory muscle strength; while buoyancy can reduce musculoskeletal load, allowing patients with poor endurance to perform aerobic exercise more comfortably (Becker, 2009). Furthermore, the warm, humid environment typically maintained in therapeutic swimming pools can reduce airway cooling and dryness during exercise, thereby lowering the likelihood of exercise-induced bronchoconstriction in susceptible individuals (Goodman and Hays, 2008). In summary, these mechanisms suggest that aquatic exercise may simultaneously improve respiratory mechanics, exercise performance, and lung function, making it a potentially effective rehabilitation strategy for asthma patients.

Over the past four decades, numerous randomized controlled trials have investigated the effects of aquatic exercise interventions in individuals with asthma; however, the findings remain inconsistent (Carson et al., 1996; Beggs et al., 2013). Some studies have reported improvements in pulmonary function parameters, including forced expiratory volume in one second (FEV_1_), peak expiratory flow (PEF), and forced vital capacity (FVC), whereas others have demonstrated limited or no significant effects compared with routine care or land-based exercise. These discrepancies may be attributed to differences in participant characteristics, age distribution, intervention modalities, exercise intensity, training duration, and outcome selection. Previous systematic reviews have primarily focused on swimming interventions, particularly among children with asthma, and have provided limited evidence regarding other forms of aquatic exercise or adult populations (Beggs et al., 2013). Although swimming represents an important component of aquatic exercise therapy, a broader range of aquatic-based interventions, including water-based exercise, aquatic training, and exercise-based hydrotherapy, are increasingly used in pulmonary rehabilitation settings (Beggs et al., 2013). Therefore, restricting analyses to swimming alone may not fully capture the potential clinical effects of aquatic exercise therapy. Furthermore, age-related differences in physiological development, airway remodeling, disease duration, and exercise adaptability may influence responses to rehabilitation interventions (Saglani and Lloyd, 2015). However, previous studies have not comprehensively evaluated whether the effects of aquatic exercise on lung function differ between pediatric and adult patients with asthma. With the accumulation of additional randomized controlled trials and the need for more clinically applicable evidence, an updated and comprehensive meta-analysis incorporating diverse aquatic exercise modalities and age-specific analyses is warranted. Therefore, this systematic review and meta-analysis aimed to comprehensively evaluate the effects of aquatic exercise therapy on lung function outcomes in patients with asthma based on randomized controlled trials. The primary outcomes were FEV_1_, FEV_1_ as a percentage of predicted value. Secondary outcomes included FEV_1_/FVC ratio, FVC, FVC as a percentage of predicted value, PEF, PEF as a percentage of predicted value, and FEF_2575_%. In addition, prespecified subgroup analyses according to age (<18 years vs ≥18 years) were performed to explore potential differences in treatment responses between pediatric and adult populations. By expanding the intervention scope beyond swimming alone and examining age-related variations in treatment effects, this study aims to provide updated evidence to guide aquatic exercise prescription and pulmonary rehabilitation strategies for individuals with asthma.

## 2 Methods

This systematic review and meta-analysis were designed, conducted, and reported in accordance with the Preferred Reporting Items for Systematic Reviews and Meta-Analyses (PRISMA) 2020 statement (Page et al., 2021). The review protocol was prospectively registered with the International Prospective Register of Systematic Reviews (PROSPERO) (Booth et al., 2011)under registration number CRD420261442630.

### 2.1 Eligibility criteria

The selection of studies is based on the PICOS (participants, intervention, control group, outcome measure, and study design) framework (Richardson et al., 1995; Chandler et al., 2019) (**Supplementary Table 1**). Studies that meet the inclusion criteria must meet the following conditions: (1) Participants: patients of any age, sex, or race who have been diagnosed with asthma according to recognized diagnostic criteria, regardless of disease severity; (2) Intervention: structured aquatic exercise performed in an aquatic environment, including swimming, aquatic exercise, aquatic fitness, aquatic training, aquatic physiotherapy, exercise therapy, aquatic aerobics, aquatic walking, aquatic jogging, aquatic resistance training, and aquatic balance training. There are no restrictions on the frequency, intensity, or duration of the intervention. For studies involving combined interventions, aquatic exercise must be the only additional intervention that distinguishes the experimental group from the control group; (3) Control group: routine care, health education, or land-based exercise. (4) Outcome measures: Studies reporting at least one pre-specified pulmonary function outcome measure, including forced expiratory volume in one second (FEV_1_), FEV_1_ (percentage of predicted value), FEV_1_/FVC (%), forced vital capacity (FVC), FVC (percentage of predicted value), peak expiratory flow (PEF), PEF (percentage of predicted value), or forced expiratory flow at 25%–75% FEF (FEF_2575_% of predicted value); and (5) Study design: randomized controlled trial (RCT).

Studies meeting any of the following criteria will be excluded: (1) non-randomized or quasi-randomized studies, observational studies, case reports, conference abstracts, reviews, editorials, letters, study protocols, or animal experiments; (2) duplicate publications or secondary reports from the same study population, in which case only the publication containing the most complete data or the longest follow-up period will be included; (3) studies with insufficient data to extract or calculate effect estimates. Or (4) Studies that cannot distinguish the independent effects of aquatic exercise from the effects of other interventions.

### 2.2 Information sources

We conducted a comprehensive literature search in PubMed, Embase, Web of Science Core Collection, Cochrane Library, and CINAHL databases, covering the period from database inception to July 4, 2026. The search strategy combined controlled thesaurus (e.g., the Medical Subject Headings [MeSH]) with free text terms related to aquatic exercise and asthma and was tailored to the indexing systems and search syntax of each database. No restrictions were placed on publication during the search (Chandler et al., 2019).

To ensure the completeness of evidence to the greatest extent possible, we manually screened the reference lists of all included studies and related review articles to identify other studies that might have been missed during the electronic database search. Full search strategies for all databases are provided in **Supplementary Table 2**.

The literature search was conducted independently by two reviewers (Xianhui Liao and Jinsong Luo). Any disagreements were resolved through discussion until a consensus was reached (Page et al., 2021).

### 2.3 Search strategy

The search strategy was developed based on the PICOS framework to maximize the sensitivity and comprehensiveness of the literature search. Controlled vocabulary (e.g., Medical Subject Headings [MeSH] in PubMed) was combined with free-text terms, and equivalent subject headings and database-specific indexing terms were applied where appropriate to accommodate the search requirements of each electronic database (Chandler et al., 2019).

The search strategy incorporated two core concepts: (1) aquatic exercise and (2) asthma. Search terms related to aquatic exercise included aquatic therapy, aquatic exercise, water-based exercise, water exercise, hydrotherapy, aquatic training, aquatic rehabilitation, water-based rehabilitation, pool exercise, water aerobics, water walking, aqua jogging, aquatic resistance exercise, aquatic balance training, swimming, and related terms. Terms describing the target condition included asthma and asthmatic. Synonymous terms within each concept were combined using the Boolean operator OR, and the two concept blocks were subsequently combined using AND. The search syntax and indexing terms were adapted as necessary for each database to ensure optimal retrieval of relevant studies. The complete search strategies for all electronic databases are provided in **Supplementary Table 2**.

### 2.4 Study selection

The study screening process was conducted using Covidence systematic review software and followed pre-defined inclusion criteria (Kellermeyer et al., 2018). Two reviewers (Xianhui Liao and Jinsong Luo) independently screened the titles and abstracts of all retrieved records after removing duplicates to identify potentially eligible studies (Page et al., 2021). Subsequently, the two reviewers independently evaluated the full text of potentially relevant articles to determine final inclusion eligibility. Any disagreements between the two reviewers during the screening process were first resolved through discussion. If no consensus could be reached, a third reviewer was consulted to adjudicate and make the final decision (Xiaofeng Cheng). The entire study screening process was reported according to the Preferred Reporting Items for Systematic Reviews and Meta-analyses (PRISMA) 2020 guidelines and presented in the form of a PRISMA flowchart.

### 2.5 Data collection process and data extraction

Data extraction was performed independently by two reviewers using standardized data extraction forms (Xianhui Liao and Jinsong Luo). Extracted information included study characteristics (first author, publication year, country, and study design), participant characteristics (sample size, age, sex, and asthma-related characteristics), details of the intervention (type of water exercise, training frequency, duration, and duration of each training session), control group characteristics, outcome measures, and relevant quantitative data required for meta-analysis. For lung function outcome measures, baseline and post-intervention values, changes, means, standard deviations, and sample size were extracted whenever possible. If the outcome measures reported in the study used different units or measurement formats, the data were converted to a comparable format before statistical analysis.Any disagreements arising during data extraction were resolved through discussion between the two reviewers; unresolved disagreements were adjudicated by a third reviewer (Xiaofeng Cheng).

### 2.6 Quality assessment

Two reviewers (Xianhui Liao and Jinsong Luo) independently assessed the risk of bias in the included randomized controlled trials using the Cochrane Risk of Bias Assessment Tool (RoB 1.0) (Higgins et al., 2011). The assessment included seven aspects: (1) randomization sequence generation, (2) allocation concealment, (3) blinding of participants and researchers, (4) blinding of outcome assessment, (5) incomplete outcome data, (6) selective reporting, and (7) other sources of bias. Each aspect was rated as low risk of bias, high risk of bias, or uncertain risk of bias, according to the criteria described in the Cochrane Manual of Interventions for Systematic Reviews. Disagreements between the two reviewers were resolved through discussion, with a third reviewer consulted as needed (Xiaofeng Cheng).

The risk of bias assessment was performed using Review Manager (RevMan) version 5.4, and the results are presented in the form of a risk of bias summary chart and graphs (Schmidt et al., 2019).

### 2.7 Data synthesis

Quantitative data were meta-analyzed using Review Manager (RevMan) version 5.4. Since all pulmonary function parameters were reported using comparable measurement scales, continuity parameters were analyzed using the mean difference (MD) and its 95% confidence interval (Beggs et al., 2013) (Chandler et al., 2019). Data were converted to uniform units where necessary before statistical pooling. All meta-analyses employed random-effects models to account for expected clinical and methodological heterogeneity among included studies, including differences in participant characteristics, aquatic exercise protocols, intervention duration, and control conditions. Statistical heterogeneity was assessed using Cochran’s Q test and the I² statistic (Higgins and Thompson, 2002). I² values ​​of approximately 25%, 50%, and 75% were considered to represent low, moderate, and high heterogeneity, respectively.

Meta-analyses were performed for each pre-specified lung function indices, including FEV_1_, FEV_1_ (percentage of predicted), FEV_1_/FVC (%), FVC, FVC (percentage of predicted), PEF, PEF (percentage of predicted), and FEF_2575_% (percentage of predicted). Indices were included in quantitative meta-analyses only when data from at least three independent studies were available.

To explore potential sources of heterogeneity, pre-specified subgroup analyses were performed. Specifically, age-based subgroup analyses were conducted for FEV_1_ and PEF, dividing participants into children and adolescents (<18 years) and adults (≥18 years). Subgroup difference tests in RevMan software were used to assess differences in effects between subgroups.

Sensitivity analyses were performed by sequentially eliminating individual studies to test the robustness and stability of the pooled effect estimates.

### 2.8 Certainty of evidence assessment (GRADE)

The certainty of evidence for each primary and secondary outcome was assessed using the Graded Assessment, Development, and Evaluation of Recommendations (GRADE) methodology (Guyatt et al., 2008). The assessment was conducted independently by two reviewers (Xianhui Liao and Jinsong Luo), based on five dimensions: risk of bias, inconsistency, indirectness, imprecision, and publication bias (Guyatt et al., 2011). According to the GRADE framework, the certainty of evidence for each outcome was categorized into four levels: high, moderate, low, or very low. Randomized controlled trials initially considered to provide high certainty of evidence may have their certainty level reduced based on identified limitations, including methodological problems, significant heterogeneity, indirectness of evidence, wide confidence intervals, or potential publication bias.

Any disagreement among reviewers regarding the certainty of evidence rating was resolved through discussion, with consultation with a third reviewer as necessary (Xiaofeng Cheng). An Outcomes Summary Table, generated using the GRADEpro Guideline Development Tool (GRADEpro GDT), summarized the overall certainty of evidence.

## 3 Results

### 3.1 Search process

**Figure 1:**
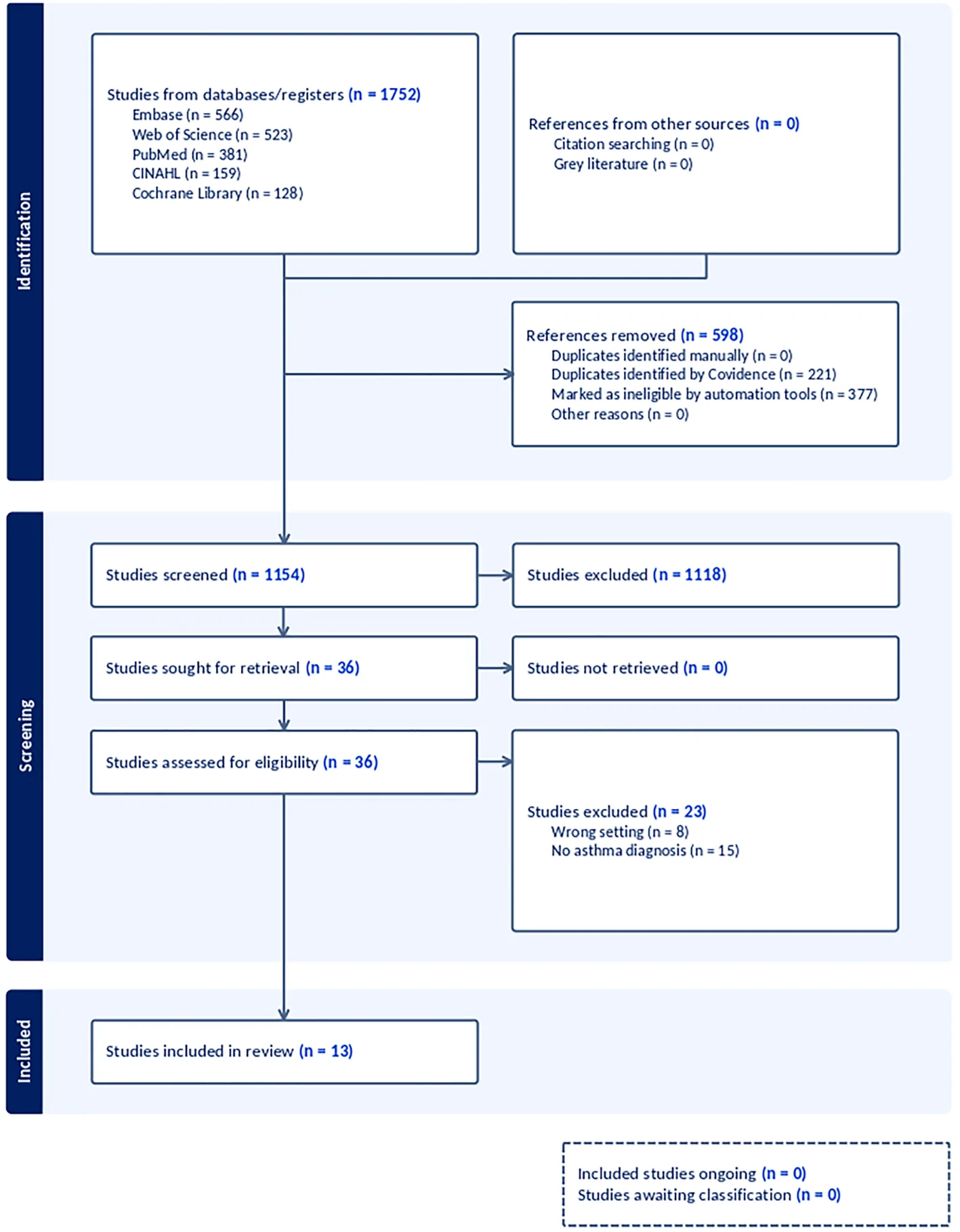
Flowchart of literature screening and inclusion for systematic review and meta-analysis.

Electronic database searches retrieved 1757 records from five databases, including PubMed (n = 381), Embase (n = 566), Web of Science (n = 523), CINAHL (n = 159), and the Cochrane Library (n = 128). No additional records were retrieved from other sources, including citation searches or grey literature searches. After removing 598 records (221 of which were duplicates identified by Covidence, and 377 were removed by automated tools), 1154 records remained for title and abstract screening. Following title and abstract screening, 1118 records were excluded, and 36 articles were retrieved for full-text evaluation. All retrieved full-text articles underwent eligibility evaluation. Of these, 23 studies were excluded, including studies conducted in unsuitable environments (n = 8) and studies on undiagnosed asthma (n = 15). Ultimately, a total of 13 randomized controlled trials (RCTs) were included in the systematic review and meta-analysis, involving 391 participants (Schnall et al., 1982; Svenonius et al., 1983; Varray et al., 1991; Emtner et al., 1998; Matsumoto et al., 1999; Weisgerber et al., 2003; Pereira, 2005; Arandelović et al., 2007; Lin et al., 2008; Wang and Hung, 2009; Weisgerber et al., 2009; Wicher et al., 2010; Carew and Cox, 2018). The complete study selection process is illustrated in the PRISMA 2020 flow diagram (**Figure 1**).

### 3.2 Description of participants

A total of 391 participants with asthma from 13 randomized controlled trials were included in this systematic review and meta-analysis. The mean age of participants across the studies included ranged from 7.3 to 36.0 years, reflecting the inclusion of both pediatric and adult populations. This age diversity allowed for subsequent subgroup analyses according to age categories. Among the included participants, 212 (54.23%) were male, 156 (39.89%) were female, while sex information was unavailable for 23 participants (5.88%) from one study. All participants were diagnosed with asthma according to the criteria reported in the original studies; however, asthma severity classifications were not consistently reported across studies. The included studies were conducted across nine countries, including China (n = 2), the United States (n = 2), Brazil (n = 2), Sweden (n = 2), Japan (n = 1), France (n = 1), Serbia (n = 1), Ireland (n = 1), and Australia (n = 1). Regarding publication language, 12 studies (92.3%) were published in English, and one study (7.7%) was published in Portuguese (**Tables 1**, **2**).

**Table 1:** General participant and study characteristics.

Characteristics	Number (%)	Mean
Total sample size	391 (100.0)	
Age, years		7.3–36.0^#^
Sex		
Male participants, n (%)	212 (54.23)	
Female participants, n (%)	156 (39.89)	
Not reported	23 (5.88) *	
Country		
China	2 (15.4)	
United States	2 (15.4)	
Brazil	2 (15.4)	
Sweden	2 (15.4)	
Japan	1 (7.7)	
France	1 (7.7)	
Serbia	1 (7.7)	
Ireland	1 (7.7)	
Australia	1 (7.7)	
Languages		
English	12 (92.3)	
Portuguese	1 (7.7)	

*Schnall et al., 1982 data on age and sex not reported.

#Mean age is presented as the range of study-specific mean ages because both pediatric and adult populations were included.

**Table 2:** Characteristics of the included studies.

First author (year, language, location)	Sample characteristics				
	T/C	Sample size	Sex	Age	Age
Weisgerber et al. (2003, English, United States)	T	5	Male/female: 3/2	8.4 ± 1.5	<18
Weisgerber et al. (2003, English, United States)	C	3	Male/female: 1/2	7.3 ± 0.6	<18
Wicher et al. (2010, English, Brasil)	T	30	Male/female: 12/18	10.35 ± 3.13	<18
Wicher et al. (2010, English, Brasil)	C	31	Male/female: 15/16	10.90 ± 2.63	<18
Matsumoto et al. (1999, English, Japan)	T	8	Male/female: 7/1	10.5 ± 0.9	<18
Matsumoto et al. (1999, English, Japan)	C	8	Male/female: 7/1	9.9 ± 1	<18
Svenonius et al. (1983, English, Sweden)	T	12	Male/female: 10/2	9-17	<18
Svenonius et al. (1983, English, Sweden)	C	10	Male/female: 6/4	9-17	<18
Varray et al. (1991, English, France)	T	7	Male/female: 6/1	11.4± 1.5	<18
Varray et al. (1991, English, France)	C	7	Male/female: 6/1	11.4± 1.8	<18
Carew et al. (2017, English, Ireland)	T	9	Male/female: 5/4	13.3 ± 1.9	<18
Carew et al. (2017, English, Ireland)	C	12	Male/female: 4/8	12.0 ± 3.1	<18
Schnall et al. (1982, English, Australia)	T	11	/	9.2 ± 3.1	<18
Schnall et al. (1982, English, Australia)	C	12	/	9.7 ± 2.3	<18
Wang et al. (2009, English, Chian)	T	15	Male/female: 10/5	10 (9–11)	<18
Wang et al. (2009, English, Chian)	C	15	Male/female: 10/5	10 (9–11)	<18
Weisgerber et al. (2009, English, United States)	T	11	Male/female: 3/8	10.8 ± 1.8	<18
Weisgerber et al. (2009, English, United States)	C	8	Male/female: 3/5	10 ± 2	<18
Lin et al. (2008, English, china)	T	16	Male/female: 11/5	10.07 ± 1.62	<18
Lin et al. (2008, English, china)	C	25	Male/female: 17/8	9.24 ± 1.61	<18
Arandelović et al. (2007, English, Serbia)	T	45	Male/female: 34/11	33.07 ± 9.81	>18
Arandelović et al. (2007, English, Serbia)	C	20	Male/female: 15/5	33.55 ± 10.88	>18
Emtner et al. (1998, English, Sweden)	T	17	Male/female: 10/7	36 ± 10	>18
Emtner et al. (1998, English, Sweden)	C	14	Male/female: 8/6	36 ± 10	>18
Pereira et al. (2005, Portuguese, Brazil)	T	20	Male/female: 5/15	34.75 ± 8.32	>18
Pereira et al. (2005, Portuguese, Brazil)	C	20	Male/female: 4/16	32.05 ± 9.23	>18

### 3.3 Description of the interventions and comparators

Thirteen randomized controlled trials included in the study investigated the effects of various forms of aquatic exercise interventions on asthma patients. These aquatic exercise programs primarily included swimming training and structured aquatic exercise interventions. Swimming was the primary intervention in studies involving children and adolescents, while aquatic exercise programs were mainly applied to adults. The characteristics of the aquatic exercise programs varied across the studies. Intervention durations ranged from 1 to 24 weeks, with exercise frequencies ranging from 1 to 6 times per week. Each exercise session lasted from 20 to 60 minutes. While most studies provided information on exercise frequency and duration, reported exercise intensity was inconsistent across studies. Control groups included a routine care group, a no-additional-exercise group, and a group engaging in other non-aquatic activities. Most control groups maintained routine asthma management and did not participate in aquatic exercise programs. One study used land-based exercise as active control, while another used non-aquatic recreational activity as a control (**Table 3**).

**Table 3:** Dosage of intervening program.

First author (year, language, location)	Dosage				
	Intervention/control	Duration (week)		Frequency (per week)	Intensity (min)
Weisgerber et al. (2003, English, United States)	swim	6	2		45
Weisgerber et al. (2003, English, United States)	control	/	/		/
Wicher et al. (2010, English, Brasil)	swim	12	2		60
Wicher et al. (2010, English, Brasil)	control	/	/		/
Matsumoto et al. (1999, English, Japan)	swim	6	6		30
Matsumoto et al. (1999, English, Japan)	control	/	/		/
Svenonius et al. (1983, English, Sweden)	swim	12-16	/		/
Svenonius et al. (1983, English, Sweden)	control	/	/		/
Varray et al. (1991, English, France)	swim	12	/		/
Varray et al. (1991, English, France)	control	/	/		/
Carew et al. (2017, English, Ireland)	swim	6	1		40
Carew et al. (2017, English, Ireland)	control	/	/		/
Schnall et al. (1982, English, Australia)	swim	10	2		50
Schnall et al. (1982, English, Australia)	Dry Land exercises	10	2		50
Wang et al. (2009, English, Chian)	swim	6	3		20
Wang et al. (2009, English, Chian)	control	/	/		/
Weisgerber et al. (2009, English, United States)	Swimming	9	3		1
Weisgerber et al. (2009, English, United States)	Golf	/	/		/
Lin et al. (2008, English, china)	swim	2day	2		20
Lin et al. (2008, English, china)	control	/	/		/
Arandelović et al. (2007, English, Serbia)	Water-based exercise	24	2		60
Arandelović et al. (2007, English, Serbia)	control	/	/		/
Emtner et al. (1998, English, Sweden)	Water-based exercise	10	5		45
Emtner et al. (1998, English, Sweden)	control	/	/		/
Pereira et al. (2005, Portuguese, Brazil)	Water-based exercise	1	1		40
Pereira et al. (2005, Portuguese, Brazil)	control	/	/		/

Overall, aquatic exercise programs exhibited significant differences, including intervention type, duration, frequency, and training characteristics.

### 3.4 Adherence, retention, and adverse events

Information regarding intervention adherence, retention, and adverse events was limited in the included studies. None of the 13 randomized controlled trials provided detailed quantitative data on adherence, such as attendance, completion rates, or adherence to the prescribed water sports program. Similarly, information on participant retention and follow-up assessments was insufficiently reported. Most studies assessed outcomes immediately following the intervention, and no studies reported long-term follow-up results. Detailed information regarding dropout or loss to follow-up was also inconsistently provided. Regarding safety outcomes, none of the studies reported adverse events related to the water sports intervention (**Table 4**).

**Table 4:** Outcome indicators, adverse events, and follow-ups included in the studies.

First author (year, language, location)	Outcome	Adverse events (yes/no/no report)	Follow-up (yes/no/no report)
Weisgerber et al. (2003, English, United States)	FEV_1_, FEV (%), FVC, FVC (%), PEF (L/s), PEF (%), FEF_25–75_%, FEF_25–75_% (%), Asthma symptoms (total score)	No report	No report
Weisgerber et al. (2003, English, United States)			
Wicher et al. (2010, English, Brasil)	PC20 (mg/mL), FVC, FEV_1_, FEV_1_/FVC ratio, FVC (%), FEV_1_ (%), FEV_1_/FVC ratio (%), FEF _25-75_% (%)	No report	No report
Wicher et al. (2010, English, Brasil)			
Matsumoto et al. (1999, English, Japan)	EIB	No report	No report
Matsumoto et al. (1999, English, Japan)			
Svenonius et al. (1983, English, Sweden)	VC, Tlc, FRC, RV, FEV_1_, FEV%, LCI, WOV, VTG, VTG/TLC%, VTG/VC%	No report	No report
Svenonius et al. (1983, English, Sweden)			
Varray et al. (1991, English, France)	FVC, FEV_1_, FEV_1_/FVC, MEF50, FEF_25-75_, FEF_25-75_/FVC	No report	No report
Varray et al. (1991, English, France)			
Carew et al. (2017, English, Ireland)	FVC (%), FEV_1_ (%), FEV_1_/FVC (%), PEF (%)	No report	No report
Carew et al. (2017, English, Ireland)			
Schnall et al. (1982, English, Australia)	FEV, FEF _25-72_ (L/sec)	No report	No report
Schnall et al. (1982, English, Australia)			
Wang et al. (2009, English, Chian)	FVC (%), FEV_1_ (%), FEV_1_/FVC (%), FEF_50_ (%)	No report	No report
Wang et al. (2009, English, Chian)			
Weisgerber et al. (2009, English, United States)	CT12, VO2max, ET,	No report	No report
Weisgerber et al. (2009, English, United States)			
Lin et al. (2008, English, china)	Exercise self-efficacy, Stages of exercise behavior change	No report	No report
Lin et al. (2008, English, china)			
Arandelović et al. (2007, English, Serbia)	FVC, FEV_1_ (L/s), FEV_1_/FVC, PEF (L/S)	No	No report
Arandelović et al. (2007, English, Serbia)			
Emtner et al. (1998, English, Sweden)	FEV_1_, PFE	No report	No report
Emtner et al. (1998, English, Sweden)			
Pereira et al. (2005, Portuguese, Brazil)	FEV_1_, PFE	No report	No report
Pereira et al. (2005, Portuguese, Brazil)			

CT12, cardiopulmonary exercise test at 12 weeks; EIB, exercise-induced bronchoconstriction; ET, exercise tolerance; FEF_2575_%, forced expiratory flow between 25% and 75% of forced vital capacity; FEV_1_, forced expiratory volume in one second; FRC, functional residual capacity; FVC, forced vital capacity; LCI, lung clearance index; MEF50, maximal mid-expiratory flow at 50% of forced vital capacity; PC20, provocative concentration causing a 20% fall in FEV_1_; PEF, peak expiratory flow; PFE, peak flow expiratory; RV, residual volume; TLC, total lung capacity; VC, vital capacity; VO_2_max, maximal oxygen uptake; VTG, trapped gas volume; VTG/TLC, trapped gas volume to total lung capacity ratio; VTG/VC, trapped gas volume to vital capacity ratio; WOV, ventilation inhomogeneity.

However, because most studies did not describe adverse event monitoring and reporting procedures, results without reported adverse events should be interpreted with caution.

### 3.5 Risk of bias

The risk of bias of the 13 included randomized controlled trials was assessed using the Cochrane Risk of Bias tool (RoB 1.0). Overall, the included studies demonstrated variable methodological quality, with most studies having unclear risk of bias in several domains due to insufficient reporting of methodological details.

**Figure 2:**
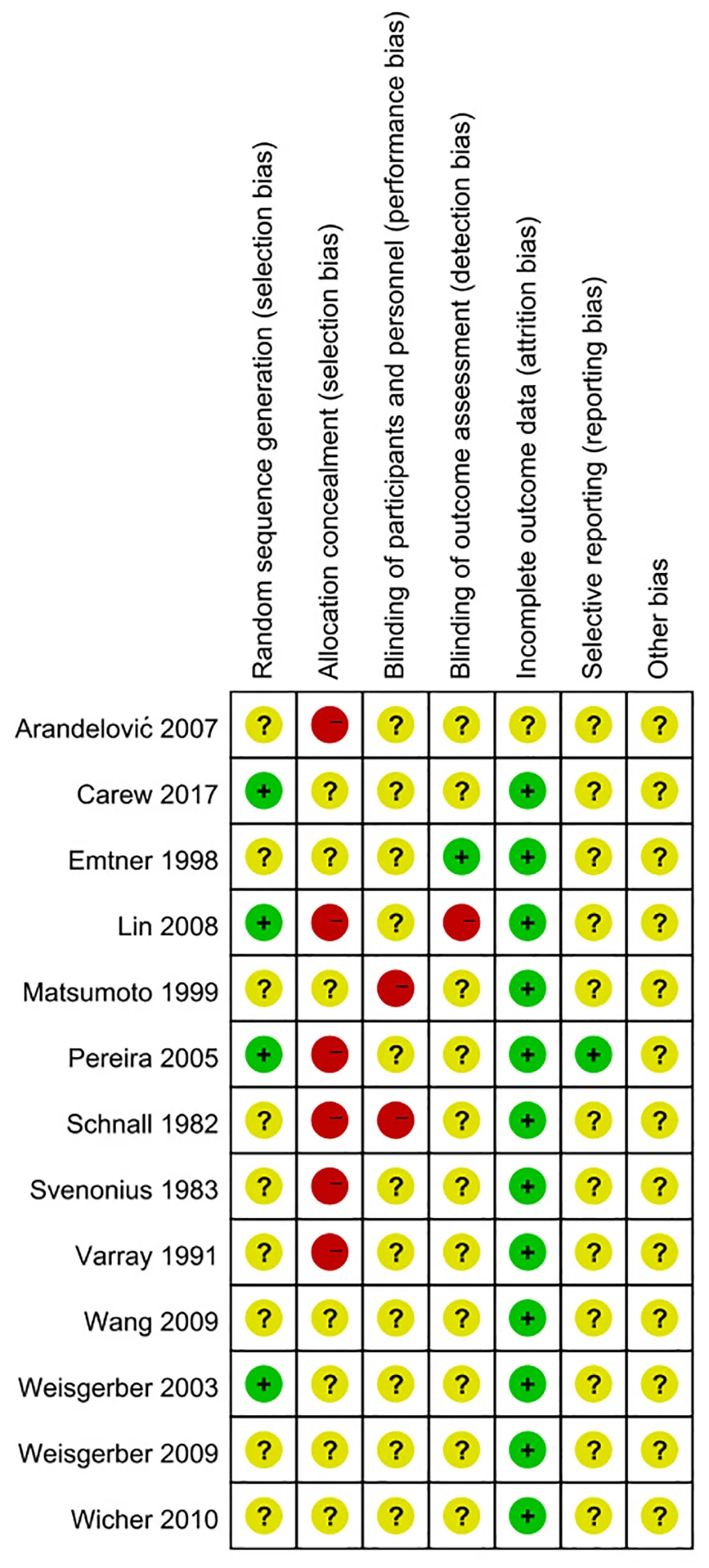
Risk of bias summary.

**Figure 3:**
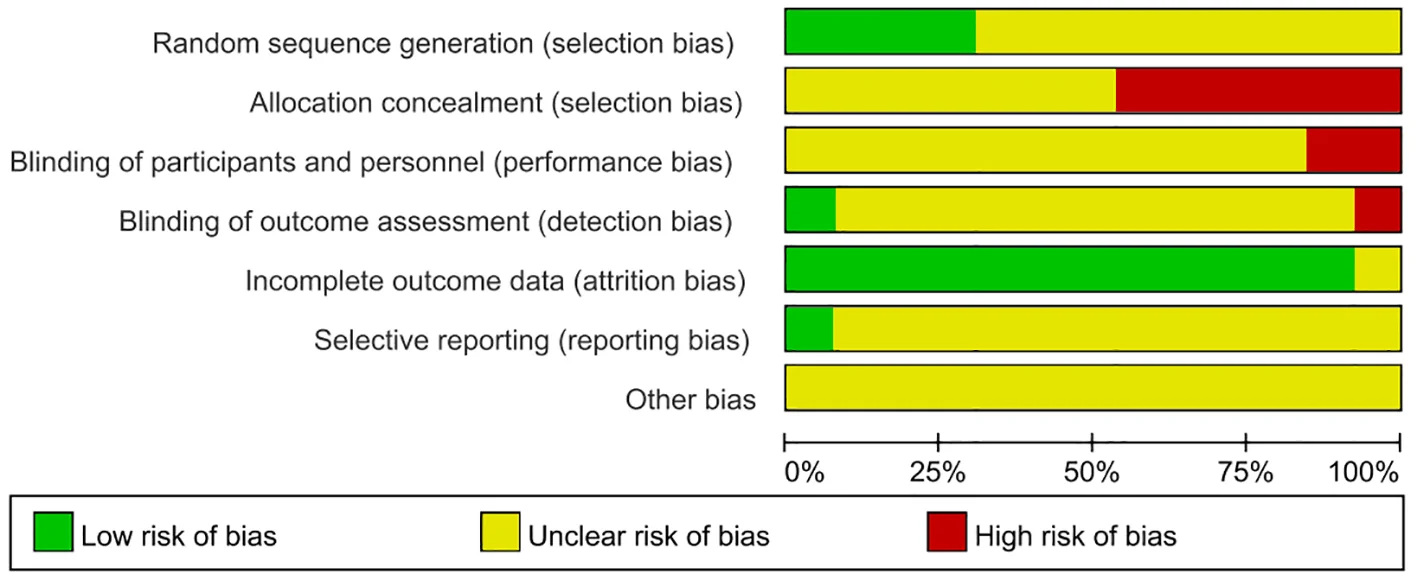
Risk of bias graph.

For random sequence generation, four studies were judged as having a low risk of bias, while the remaining nine studies were classified as having an unclear risk because the methods used to generate the random sequence were not adequately described. Regarding allocation concealment, five studies were considered to have a high risk of bias, and eight studies were judged as having an unclear risk, with no studies providing sufficient information to be classified as low risk. Due to the nature of aquatic exercise interventions, blinding of participants and personnel was difficult to achieve. One study was judged as having a low risk, two studies as having a high risk, and ten studies as having an unclear risk for performance bias. For blinding of outcome assessment, one study was considered low risk, one study was considered high risk, and the remaining eleven studies were classified as unclear risk. Most studies adequately reported participant retention and outcome data. For incomplete outcome data, twelve studies were judged as having a low risk of bias, whereas one study was considered unclear. Selective reporting was generally difficult to determine because trial protocols or prespecified analysis plans were unavailable; one study was judged as low risk and twelve studies were classified as unclear risk. All studies were considered to have an unclear risk of other bias due to insufficient information regarding additional sources of bias. The detailed risk of bias assessment is presented in **Figures 2**, **3**.

### 3.6 Primary outcomes

#### 3.6.1 Forced expiratory volume in one second

A total of six randomized controlled trials involving 228 participants contributed data to the meta-analysis of FEV_1_. Compared with control conditions, aquatic exercise therapy resulted in a statistically significant improvement in FEV_1_ (MD = 0.21 L, 95% CI 0.02 to 0.41; P = 0.03; I² = 34%), indicating a beneficial effect of aquatic exercise on expiratory airflow capacity.

**Figure 4:**
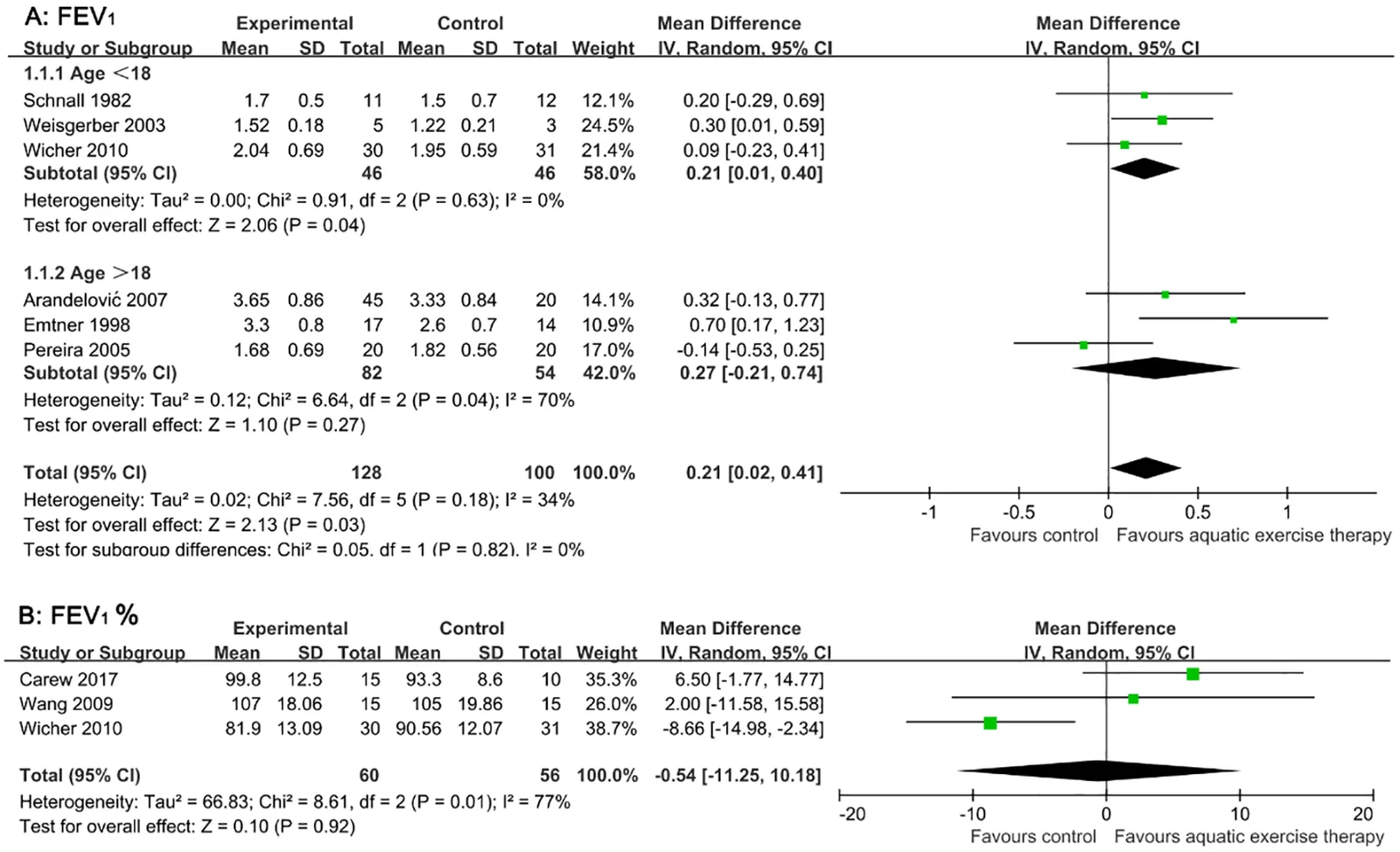
Forest plots of the effects of aquatic exercise therapy on forced expiratory volume in one second (FEV_1_) and FEV_1_ (% predicted) in patients with asthma. **(A)** FEV_1_. **(B)** FEV_1_ (% predicted).

Subgroup analysis according to age demonstrated different effects between pediatric and adult populations. Among participants aged <18 years, aquatic exercise significantly improved FEV_1_ (MD = 0.21 L, 95% CI 0.01 to 0.40; P = 0.04; I² = 0%). In contrast, among participants aged ≥18 years, the effect was not statistically significant (MD = 0.27 L, 95% CI −0.21 to 0.74; P = 0.27; I² = 70%). The test for subgroup differences showed no statistically significant interaction between age groups (P = 0.82) (**Figure 4A**; **Supplementary Table 3**).

#### 3.6.2 FEV_1_ (% predicted)

Three studies involving 116 participants reported FEV_1_ (% predicted). The pooled analysis showed no significant difference between aquatic exercise and control groups (MD = −0.54%, 95% CI −11.25 to 10.18; P = 0.92; I² = 77%) (**Figure 4B**; **Supplementary Table 3**).

Although individual studies showed variation in effect estimates, the overall pooled result did not demonstrate evidence that aquatic exercise significantly altered FEV_1_ expressed as a percentage of predicted values.

### 3.7 Secondary outcomes

#### 3.7.1 FEV_1_/FVC (%)

**Figure 5:**
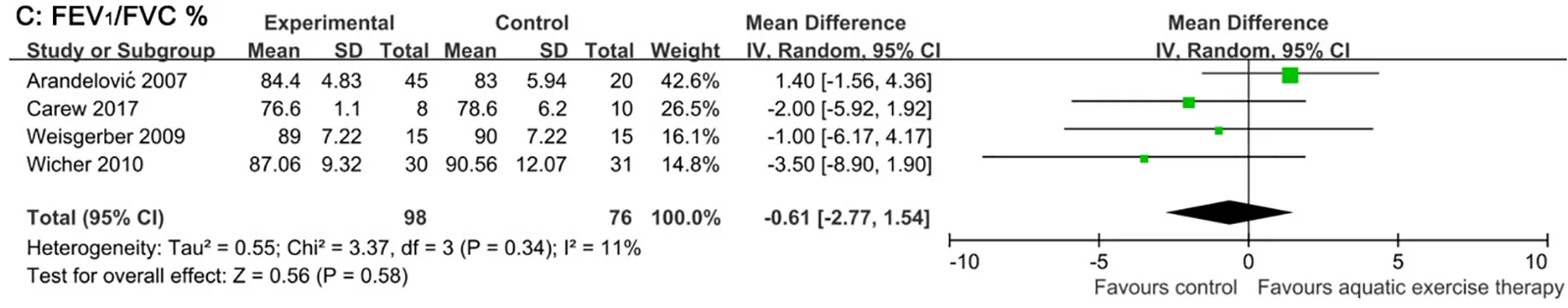
Forest plot of the effects of aquatic exercise therapy on FEV_1_/FVC (%) in patients with asthma. **(C)** FEV1/FVC (%).

Four studies including 174 participants evaluated the effect of aquatic exercise on FEV_1_/FVC (%). No statistically significant improvement was observed following aquatic exercise compared with controls (MD = −0.61%, 95% CI −2.77 to 1.54; P = 0.67; I² = 0%) (**Figure 5C**; **Supplementary Table 3**).

The pooled estimate showed low heterogeneity across studies, suggesting consistent but non-significant effects among included trials.

#### 3.7.2 Forced vital capacity

**Figure 6:**
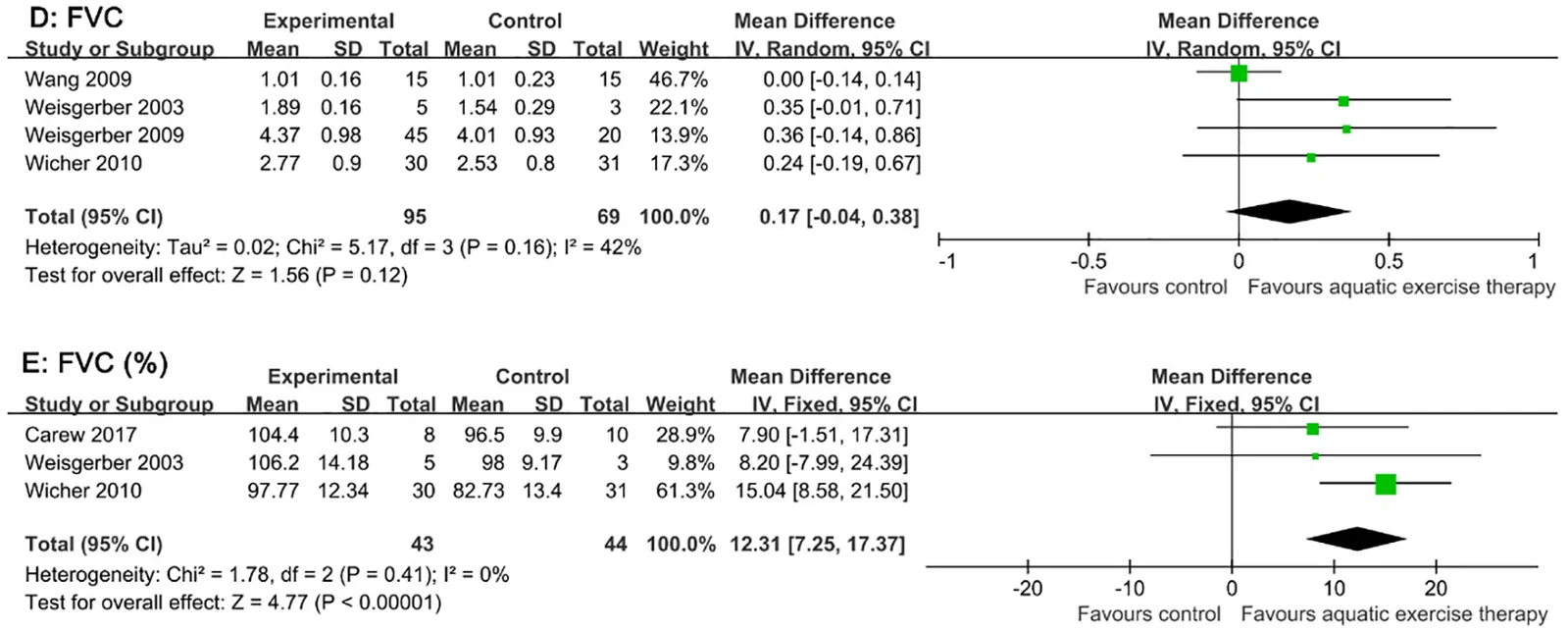
Forest plots of the effects of aquatic exercise therapy on forced vital capacity outcomes in patients with asthma. **(D)** FVC. **(E)** FVC (% predicted).

Four studies including 164 participants, provided data on absolute FVC values. The meta-analysis showed that aquatic exercise did not significantly improve FVC compared with control conditions (MD = 0.17 L, 95% CI -0.04 to 0.38; P = 0.12; I² = 42%) (**Figure 6D**; **Supplementary Table 3**).

Although the pooled effect favored aquatic exercise, the confidence interval crossed the line of no effect.

#### 3.7.3 Forced vital capacity (% predicted)

Three studies involving 87 participants reported FVC (% predicted). Aquatic exercise therapy significantly improved FVC (% predicted) compared with control groups (MD = 12.32%, 95% CI 7.25 to 17.37; P = 0.001; I² = 0%) (**Figure 6E**; **Supplementary Table 3**).

The pooled estimate indicated a consistent beneficial effect, with no evidence of statistical heterogeneity across studies.

#### 3.7.4 Peak expiratory flow

Four studies involving 144 participants reported the effect of aquatic exercise therapy on PEF. Compared with control conditions, aquatic exercise resulted in a statistically significant improvement in PEF (MD = 0.51 L/s, 95% CI 0.12 to 0.91; P = 0.01; I² = 0%).

**Figure 7:**
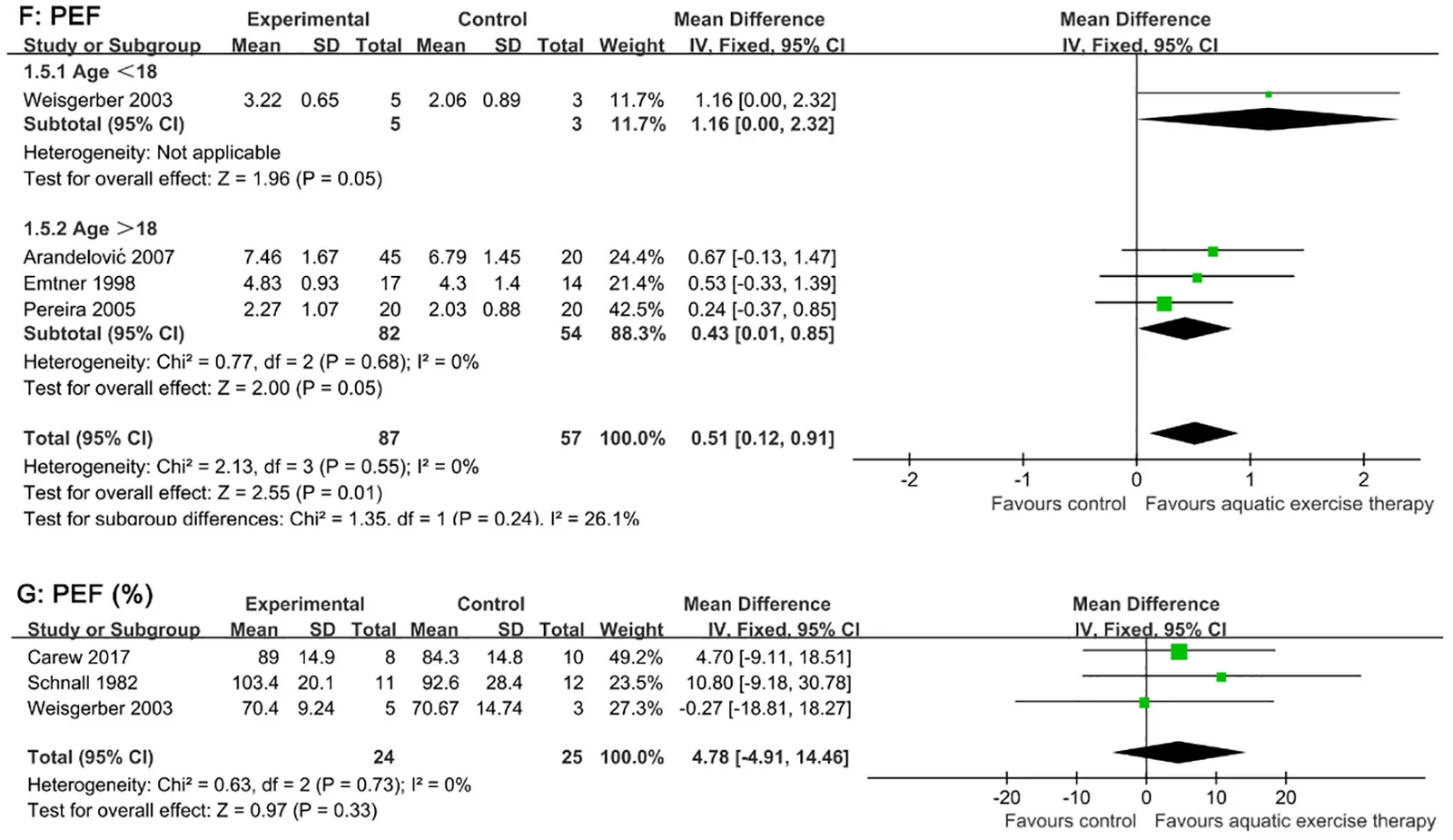
Forest plots of the effects of aquatic exercise therapy on peak expiratory flow outcomes in patients with asthma. **(F)** PEF. **(G)** PEF (% predicted).

Subgroup analysis according to age showed that aquatic exercise had a positive effect in both pediatric and adult populations. Among participants aged <18 years, the pooled effect estimate indicated a statistically significant improvement (MD = 1.16 L/s, 95% CI 0.00 to 2.32; P = 0.05). Similarly, participants aged ≥18 years demonstrated a borderline statistically significant improvement in PEF (MD = 0.43 L/s, 95% CI 0.01 to 0.85; P = 0.05; I² = 0%). No significant difference was detected between the age subgroups (P for subgroup difference = 0.24) (**Figure 7F**; **Supplementary Table 3**).

#### 3.7.5 Peak expiratory flow (% predicted)

Three studies involving 49 participants assessed PEF (% predicted). No statistically significant difference was observed between aquatic exercise and control groups (MD = 4.78%, 95% CI −4.91 to 14.46; P = 0.33; I² = 0%) (**Figure 7G**; **Supplementary Table 3**).

The pooled analysis suggested that aquatic exercise did not significantly influence PEF expressed as a percentage of predicted values.

#### 3.7.6 Forced expiratory flow at 25–75% of forced vital capacity (FEF_2575_%)

**Figure 8:**
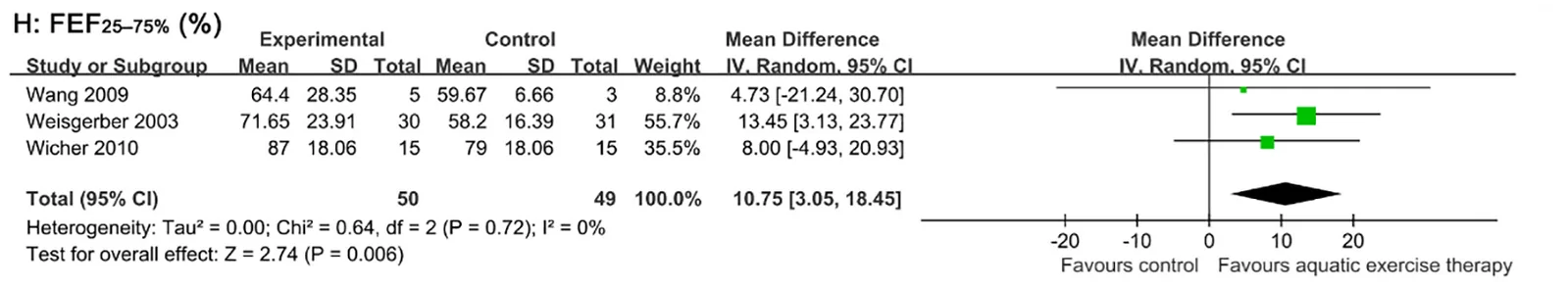
Forest plot of the effect of aquatic exercise therapy on forced expiratory flow at 25–75% of forced vital capacity (FEF25–75%) in patients with asthma. **(H)** FEF25–75%.

Three studies including 99 participants evaluated the effect of aquatic exercise on FEF_2575_%. The pooled analysis demonstrated a significant improvement in FEF_2575_% following aquatic exercise compared with control conditions (MD = 10.75%, 95% CI 3.05 to 18.45; P = 0.006; I² = 0%) (**Figure 8H**; **Supplementary Table 3**).

The low heterogeneity among studies suggested consistent effects across the included trials.

### 3.8 Publication bias

We used funnel plots to assess publication bias for reported lung function parameters. For parameters with enough included studies, we performed a visual check for funnel plot asymmetry. For the primary parameter FEV_1_, we included 6 studies for funnel plot assessment. The funnel plots did not show significant asymmetry, suggesting no significant publication bias.

**Figure 9:**
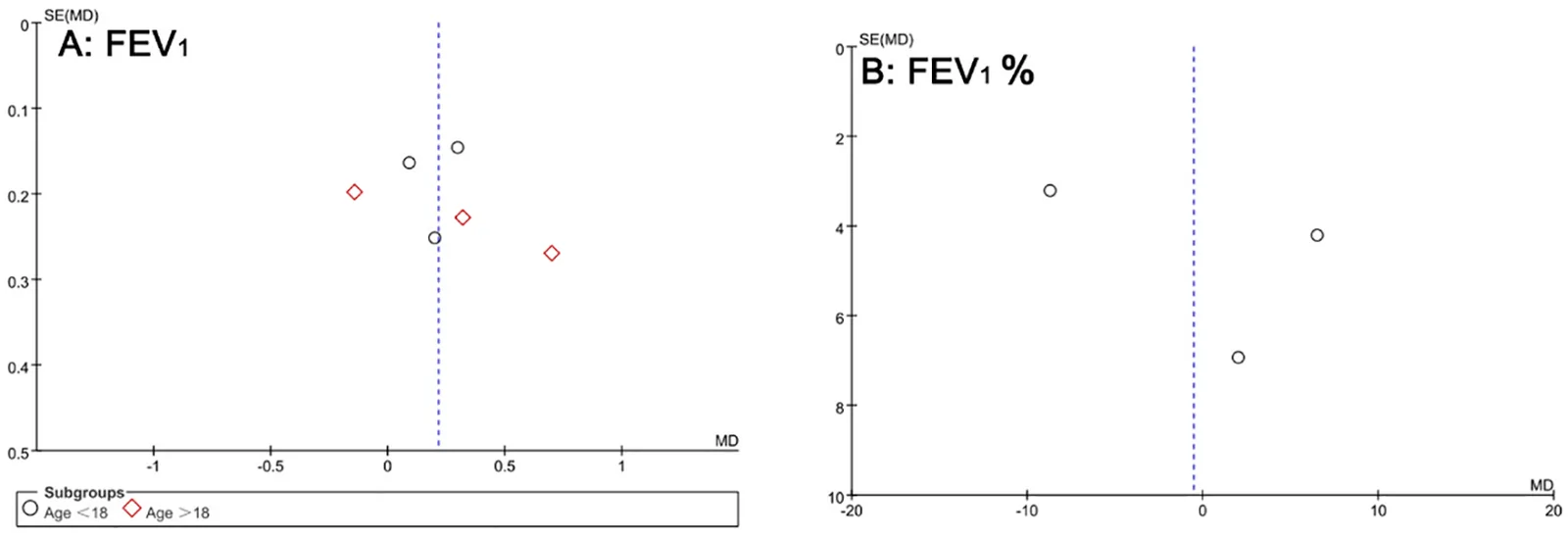
Funnel plots assessing potential publication bias for primary pulmonary function outcomes in patients with asthma. **(A)** FEV_1_; **(B)** FEV_1_ (% predicted).

**Figure 10:**
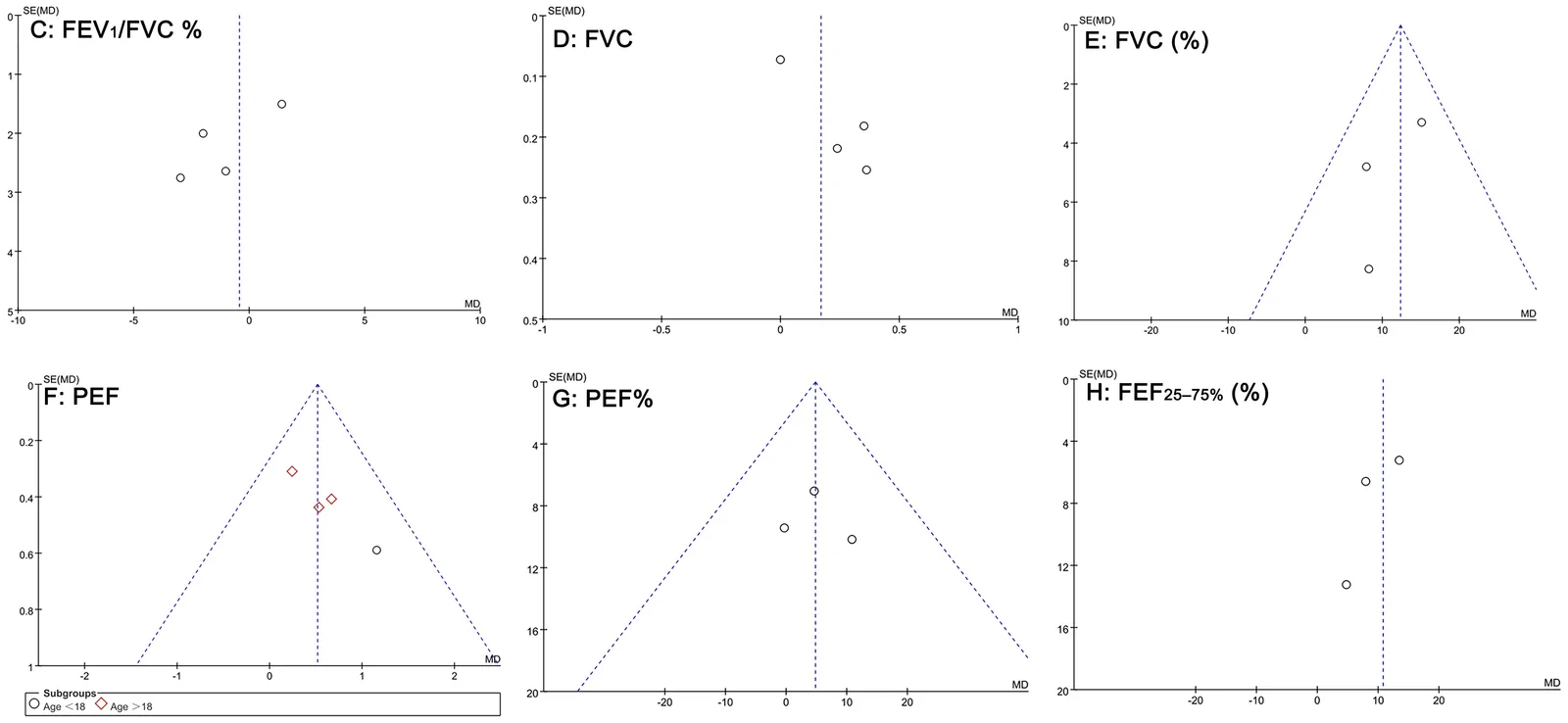
Funnel plots assessing potential publication bias for secondary pulmonary function outcomes in patients with asthma. **(C)** FEV₁/FVC (%); **(D)** FVC; **(E)** FVC (% predicted); **(F)** PEF; **(G)** PEF (% predicted); **(H)** FEF₂₅–₇₅ (%).

For the remaining parameters, including FEV_1_ (percentage of predicted value), FEV_1_/FVC (%), PEF, PEF (percentage of predicted value), FEF_2575_%, FVC (percentage of predicted value), and FVC, funnel plot analyses were not performed because the number of included studies was insufficient (<10 studies). The corresponding funnel plots are shown in **Figures 9**, **10**.

### 3.9 Certainty of evidence (GRADE)

The certainty of evidence for each pulmonary function measure was assessed using the GRADE method (**Table 5**). Overall, the certainty of evidence for the assessed measures ranged from very low to moderate. The certainty of evidence for PEF, FEF_2575_%, and FVC (percentage of predicted value) was moderate, indicating a relatively consistent beneficial effect of aquatic exercise on these measures, but some limitations remained due to methodological issues in the included studies. The certainty of evidence for FEV_1_ and FVC was low, primarily due to the risk of bias and insufficient precision in effect estimation. Although aquatic exercise significantly improved FEV_1_, confidence in the extent of the efficacy remained low. The certainty of evidence for FEV_1_ (percentage of predicted value), FEV_1_/FVC (%), and PEF (percentage of predicted value) was very low. These results were downgraded due to methodological limitations, inconsistencies in study results, and/or insufficient precision in pooled estimates (including confidence intervals crossing the no-effect line).

**Table 5:** Certainty of evidence assessment using the GRADE approach.

Certainty assessment							№ of patients		Effect		Certainty	Importance
№ of studies	Study design	Risk of bias	Inconsistency	Indirectness	Imprecision	Other considerations	[intervention]	[comparison]	Relative (95% CI)	Absolute (95% CI)		
FEV1												
6	randomised trials	serious^a^	serious^b^	not serious	not serious	none	128	100	–	MD 0.21 higher (0.02 higher to 0.41 higher)	⨁⨁◯◯ Low^a^,^b^	CRITICAL
FEV1%												
3	randomised trials	serious^a^	serious^b^,^c^	not serious	serious^d^	none	60	56	–	MD 0.54 lower (11.25 lower to 10.18 higher)	⨁◯◯◯ Very low^a^,^b^,^c^,^d^	CRITICAL
FEV1/FVC (%)												
4	randomised trials	serious^a^	serious^b^	not serious	serious^d^	none	98	76	–	MD 0.61 lower (2.77 lower to 1.54 higher)	⨁◯◯◯ Very low^a^,^b^,^d^	CRITICAL
FVC												
4	randomised trials	serious^a^	not serious	not serious	serious^d^	none	95	69	–	MD 0.17 higher (0.04 lower to 0.38 higher)	⨁⨁◯◯ Low^a^,^d^	CRITICAL
FVC (%)												
3	randomised trials	serious^a^	not serious	not serious	not serious	none	43	44	–	MD 12.32 higher (7.25 higher to 17.37 higher)	⨁⨁⨁◯ Moderate^a^	CRITICAL
PEF												
4	randomised trials	serious^a^	not serious	not serious	not serious	none	87	57	–	0.51 higher (0.12 higher to 0.91 higher)	⨁⨁⨁◯ Moderate^a^	CRITICAL
PEF (%)												
3	randomised trials	serious^a^	serious^b^	not serious	serious^d^	none	24	25	–	MD 4.78 higher (4.91 lower to 14.46 higher)	⨁◯◯◯ Very low^a^,^b^,^d^	CRITICAL
FEF25-75% (%)												
3	randomised trials	serious^a^	not serious	not serious	not serious	none	50	49	–	MD 10.75 higher (3.05 higher to 18.45 higher)	⨁⨁⨁◯ Moderate^a^	CRITICAL

CI, confidence interval; MD, mean difference; a: Some concerns or high risk of bias in included studies; b: The study results show inconsistencies; c: The results of the study show a high degree of heterogeneity; d: The confidence interval crosses the line of no effect (0 for MD), indicating uncertainty in the estimate.

Overall, while aquatic exercise therapy has shown potential benefits for some lung function parameters, the certainty of the evidence remains limited, and more high-quality randomized controlled trials with larger sample sizes and more standardized intervention protocols are needed to confirm these findings.

## 4 Discussion

To our knowledge, this systematic review and meta-analysis represent the first comprehensive evaluation of the effects of aquatic exercise therapy on objective lung function outcomes in patients with asthma across both pediatric and adult populations. Unlike previous reviews that primarily focused on swimming interventions among children with asthma (Carson et al., 1996; Beggs et al., 2013), this study expanded the intervention scope to include a broader range of aquatic-based exercise modalities, including swimming, water-based exercise, aquatic training, and exercise-based hydrotherapy. Furthermore, this review is the first to explore potential age-related differences in the effects of aquatic exercise therapy through prespecified subgroup analyses. This meta-analysis included 13 randomized controlled trials involving 391 asthma patients. Overall, aquatic exercise therapy was associated with a significant improvement in forced expiratory volume in one second (FEV_1_), indicating a beneficial effect on dynamic expiratory function. However, no statistically significant improvements were observed in FEV_1_ (percentage of predicted value), suggesting that the effects of aquatic exercise may be more reflected in absolute expiratory volume than in standardized pulmonary function parameters. Age-stratified analysis further showed that improvements in FEV_1_ were statistically significant in participants under 18 years of age, but not in adults. These findings suggest that younger asthma patients may derive greater pulmonary function benefits from aquatic exercise intervention (Saglani and Lloyd, 2015). Among secondary outcomes, aquatic exercise therapy significantly improved several measures of pulmonary function, including peak expiratory flow (PEF), forced vital capacity expressed as a percentage of predicted value (FVC%), and forced expiratory flow between 25% and 75% of forced vital capacity (FEF_2575_%). In contrast, no statistically significant improvement was observed in FEV_1_/FVC (%), absolute FVC, or PEF%. Collectively, these findings suggest that aquatic exercise may preferentially influence measures related to expiratory airflow and ventilatory performance rather than inducing broad improvements across all pulmonary function parameters (Pellegrino et al., 2005). The discrepancies observed between absolute pulmonary function values and percentage predicted values warrant careful interpretation. Although these measures are derived from the same pulmonary function parameters, the corresponding meta-analyses were based on different subsets of randomized controlled trials. For example, FEV_1_ (L) and FEV_1_ (% predicted) were reported by different numbers of studies with different sample sizes and participant characteristics. Similar differences were observed regarding PEF and FVC outcomes. Therefore, variations in age distribution, baseline pulmonary function, disease characteristics, reference equations used to calculate predicted values, and intervention protocols may have contributed to the inconsistent statistical significance between absolute and percentage predicted outcomes. These findings highlight the importance of considering clinical and methodological heterogeneity when interpreting pulmonary function outcomes.

Previous studies have primarily investigated the effects of swimming interventions in children with asthma, with limited evidence regarding other aquatic exercise modalities or adult populations (Beggs et al., 2013). Previous reviews have suggested that swimming may improve exercise capacity and some aspects of pulmonary function; however, their conclusions were restricted by the inclusion of pediatric populations and the narrow definition of aquatic exercise (Carson et al., 1996). This meta-analysis expands upon previous research evidence in several important ways. First, we included both children and adults with asthma, allowing for a broader evaluation of aquatic exercise therapy across different age groups. Second, we focused not only on swimming but also on a variety of aquatic exercise modalities, such as water-based exercises, aquatic training, and exercise therapy. This broader approach reflects the diverse aquatic rehabilitation protocols used in clinical practice. Third, we conducted age-based subgroup analyses, enabling us to explore potential differences in treatment response between children and adults. Our results indicate that aquatic exercise therapy significantly improved FEV_1_, but no significant effect was observed on the percentage of FEV_1_ to predicted value. This suggests that aquatic exercise may have a greater impact on absolute expiratory volume and dynamic ventilation capacity than on standardized indicators reflecting airway obstruction (Graham et al., 2019). The differences in outcomes may be related to baseline lung function, disease duration, and the ability of exercise intervention to improve functional performance, rather than underlying airway pathology (Welsh et al., 2005).

Several mechanisms may explain the potential benefits of aquatic exercise therapy for asthma. First, immersion in water creates hydrostatic pressure on the chest wall, increasing respiratory load and promoting respiratory muscle activation (Becker, 2009). Repeated exposure to this physiological challenge may enhance respiratory muscle endurance and improve ventilation efficiency. Second, the buoyancy of water reduces mechanical load, allowing individuals to perform aerobic exercise with lower subjective effort (Becker, 2009). This property may be particularly beneficial for patients with exercise intolerance, improving their adherence and sustained participation compared to certain land-based activities (Eichenberger et al., 2013). Third, regular aerobic exercise can improve cardiopulmonary function, reduce ventilation requirements during daily activities, and may influence asthma-related inflammatory processes. Although the included studies did not assess inflammatory markers, previous research suggests that exercise-induced physiological adaptations may contribute to improved respiratory function (Lötvall et al., 2011). Furthermore, aquatic activities such as swimming require controlled breathing patterns, which may help improve respiratory coordination and control (Lahart and Metsios, 2018). This may partially explain the observed improvements in expiratory flow-related parameters, including PEF and FEF_2575_.

A key finding of this review is that treatment efficacy varies with age. Subgroup analyses showed that aquatic exercise significantly improved FEV_1_ in participants under 18 years of age, but not in adults. Several factors could explain this difference. Children and adolescents typically have greater lung development potential and airway adaptability, which may allow them to achieve greater improvements after exercise intervention (Von Mutius and Smits, 2020). In contrast, adults with asthma may have a longer disease duration, persistent airway inflammation, and altered airway structure, which could limit the extent of functional improvement (James and Wenzel, 2007; Mauad et al., 2007). However, due to the limited number of studies in the adult subgroup, statistical power may be insufficient, and results that are not statistically significant in the adult subgroup should be interpreted with caution. Notably, PEF improved in both age groups, although the results were close to the statistical significance threshold. These findings suggest that aquatic exercise may be beneficial across age groups, but the degree of improvement may vary depending on age-related physiological characteristics.

The findings of this meta-analysis suggest that aquatic exercise therapy may serve as a useful adjunctive intervention for asthma management (Bateman et al., 2008). Compared with conventional exercise modalities, aquatic exercise provides a low-impact environment that may improve exercise tolerance and facilitate participation among individuals who experience exercise-related symptoms (Becker, 2009). The observed improvements in FEV_1_ and expiratory flow parameters indicate that aquatic exercise may contribute to enhanced pulmonary functional capacity, particularly in younger patients. Therefore, structured aquatic exercise programs may be considered as part of comprehensive asthma rehabilitation strategies. Nevertheless, aquatic exercise should be complemented rather than replace guideline-based pharmacological treatment. Future clinical applications should consider individual characteristics, including age, disease severity, physical capacity, and patient preferences.

This study has several strengths. First, it is the first systematic review and meta-analysis to evaluate the effects of aquatic exercise therapy on objective lung function outcomes in both pediatric and adult patients with asthma. Secondly, the intervention was expanded from swimming to a wider range of aquatic sports. Thirdly, age-group subgroup analyses were conducted to explore potential differences in treatment response. Furthermore, the following limitations should be considered. The relatively small number of included studies and total sample size limited the statistical power of some outcome measures and limited the reliability of publication bias assessment for some outcomes. Although funnel plots were performed when sufficient studies were available, most outcomes included fewer than 10 studies; therefore, the results of funnel plot analyses should be interpreted cautiously (Chandler et al., 2019). In addition, the overall risk of bias was rated as moderate, primarily due to underreporting of randomization procedures, allocation concealment, and blinding. Significant heterogeneity existed in intervention characteristics, including exercise duration, frequency, and intensity. Furthermore, underreporting of asthma severity, medication use, and disease phenotype hampered further subgroup analyses.

Future studies should include larger, well-designed randomized controlled trials with standardized aquatic exercise protocols. Further research is needed to determine whether factors such as age, asthma phenotype, baseline lung function, and disease severity influence responses to aquatic exercise therapy.

## 5 Conclusion

In conclusion, this systematic review and meta-analysis suggest that aquatic exercise therapy may improve lung function in patients with asthma, particularly FEV_1_ and expiratory flow-related parameters. While the evidence of benefit in adult patients is still unclear, its efficacy appears to be more significant in pediatric patients due to the limited number of relevant studies. Aquatic exercise therapy may be a promising adjunctive therapy for asthma rehabilitation; however, more high-quality randomized controlled trials are needed to determine the optimal training regimen and assess its long-term clinical benefits.

## Supplementary material

Supplementary Table 2 Search strategy.
