# Supplementary material for: Effects of aquatic exercise therapy on lung function in patients with asthma: a systematic review and meta-analysis
Source: Front Physiol. 2026 Aug 28;17:1931034. doi: 10.3389/fphys.2026.1931034 (PMC13563647; doi:10.3389/fphys.2026.1931034)
Supplement: Supplementary file 1 [file DataSheet1.docx]

**Supplementary Table 1 The PICOs details**

| **Participant** | **Intervention** | **Control** | **Outcomes** | **Studies** |
| --- | --- | --- | --- | --- |
| Patients are diagnosed with asthma according to recognized diagnostic criteria, regardless of gender, race, or age. | Structured aquatic exercise interventions performed in a water environment, including swimming, aquatic exercise, water-based exercise, aquatic training, aquatic physical therapy, exercise-based hydrotherapy, water aerobics, water walking, aqua jogging, aquatic resistance exercise, and aquatic balance training. Interventions were required to involve active, supervised or prescribed physical exercise conducted in water. | One or more of the following: routine care, health education, other land sports | Primary outcomes: Lung function parameters, including FEV₁ and FEV₁ (% predicted). Secondary outcomes: FEV₁/FVC (%), FVC, FVC (% predicted), PEF, PEF (% predicted), and FEF₂₅–₇₅% (% predicted). | Randomized controlled trial (RCT) |
