# Supplementary material for: Effects of aquatic exercise therapy on lung function in patients with asthma: a systematic review and meta-analysis
Source: Front Physiol. 2026 Aug 28;17:1931034. doi: 10.3389/fphys.2026.1931034 (PMC13563647; doi:10.3389/fphys.2026.1931034)
Supplement: Supplementary file 2 [file DataSheet2.docx]

| **Certainty assessment** | | | | | | | **№ of patients** | | **Effect** | | **Certainty** | **Importance** |
| --- | --- | --- | --- | --- | --- | --- | --- | --- | --- | --- | --- | --- |
| **№ of studies** | **Study design** | **Risk of bias** | **Inconsistency** | **Indirectness** | **Imprecision** | **Other considerations** | **[intervention]** | **[comparison]** | **Relative (95% CI)** | **Absolute (95% CI)** |  |  |
| **FEV1** | | | | | | | | | | | | |
| 6 | randomised trials | serious^a^ | serious^b^ | not serious | not serious | none | 128 | 100 | - | MD **0.21 higher** (0.02 higher to 0.41 higher) | ⨁⨁◯◯ Low^a,b^ | CRITICAL |
| **FEV1%** | | | | | | | | | | | | |
| 3 | randomised trials | serious^a^ | serious^b,c^ | not serious | serious^d^ | none | 60 | 56 | - | MD **0.54 lower** (11.25 lower to 10.18 higher) | ⨁◯◯◯ Very low^a,b,c,d^ | CRITICAL |
| **FEV1/FVC (%)** | | | | | | | | | | | | |
| 4 | randomised trials | serious^a^ | serious^b^ | not serious | serious^d^ | none | 98 | 76 | - | MD **0.44 lower** (2.43 lower to 1.56 higher) | ⨁◯◯◯ Very low^a,b,d^ | CRITICAL |
| **FVC** | | | | | | | | | | | | |
| 4 | randomised trials | serious^a^ | not serious | not serious | serious^d^ | none | 95 | 69 | - | MD **0.17 higher** (0.04 lower to 0.38 higher) | ⨁⨁◯◯ Low^a,d^ | CRITICAL |
| **FVC (%)** | | | | | | | | | | | | |
| 3 | randomised trials | serious^a^ | not serious | not serious | not serious | none | 43 | 44 | - | MD **12.32 higher** (7.25 higher to 17.37 higher) | ⨁⨁⨁◯ Moderate^a^ | CRITICAL |
| **PEF** | | | | | | | | | | | | |
| 4 | randomised trials | serious^a^ | not serious | not serious | not serious | none | 87 | 57 | - | **0.51 higher** (0.12 higher to 0.91 higher) | ⨁⨁⨁◯ Moderate^a^ | CRITICAL |
| **PEF (%)** | | | | | | | | | | | | |
| 3 | randomised trials | serious^a^ | serious^b^ | not serious | serious^d^ | none | 24 | 25 | - | MD **4.78 higher** (4.91 lower to 14.46 higher) | ⨁◯◯◯ Very low^a,b,d^ | CRITICAL |
| **FEF25-75% (%)** | | | | | | | | | | | | |
| 3 | randomised trials | serious^a^ | not serious | not serious | not serious | none | 50 | 49 | - | MD **10.75 higher** (3.05 higher to 18.45 higher) | ⨁⨁⨁◯ Moderate^a^ | CRITICAL |

**CI:** confidence interval; **MD:** mean difference; **a:** Some concerns or high risk of bias in included studies; **b:** The study results show inconsistencies; **c:** The results of the study show a high degree of heterogeneity; **d:** The confidence interval crosses the line of no effect (0 for MD), indicating uncertainty in the estimate.
