# Supplementary material for: Effects of aquatic exercise therapy on lung function in patients with asthma: a systematic review and meta-analysis
Source: Front Physiol. 2026 Aug 28;17:1931034. doi: 10.3389/fphys.2026.1931034 (PMC13563647; doi:10.3389/fphys.2026.1931034)
Supplement: Supplementary file 3 [file DataSheet3.docx]

**Supplementary Table 3 Overall effects**

| **Outcome** | **95% CI, I2** | **P value** |
| --- | --- | --- |
| FEV_1_ | 0.21[0.02 to 0.41], I² = 34% | 0.03* |
| FEV_1_% | -0.54[-11.25 to 10.18], I² = 77% | 0.92 |
| FEV_1_/FVC (%) | -0.44[-2.43 to 1.56], I² = 0% | 0.67 |
| PEF | 0.51[0.12 to 0.91], I² = 0% | 0.01* |
| PEF (%) | 4.78[-4.91 to 14.46], I² = 0.0% | 0.33 |
| FEF_25-75%_ (%) | 10.75[3.05 to 18.45], I² = 0% | 0.006** |
| FVC | 0.17[-0.04 to 0.38], I² = 42% | 0.12 |
| FVC (%) | 12.32[7.25 to 17.37], I² = 0% | 0.001** |

Note: FEF₂₅–₇₅% (% predicted): Forced expiratory flow at 25–75% of forced vital capacity as a percentage of the predicted value; FEV₁: Forced expiratory volume in one second; FEV₁ (% predicted): Forced expiratory volume in one second as a percentage of the predicted value; FEV₁/FVC: Ratio of forced expiratory volume in one second to forced vital capacity; FVC: Forced vital capacity; FVC (% predicted): Forced vital capacity as a percentage of the predicted value; PEF: Peak expiratory flow; PEF (% predicted): Peak expiratory flow as a percentage of the predicted value.

Difference *: p ＜ 0.05; **: p ＜ 0.01
