# Supplementary material for: Effects of aquatic exercise therapy on lung function in patients with asthma: a systematic review and meta-analysis
Source: Front Physiol. 2026 Aug 28;17:1931034. doi: 10.3389/fphys.2026.1931034 (PMC13563647; doi:10.3389/fphys.2026.1931034)
Supplement: Supplementary file 4 [file DataSheet4.docx]

2026.7.4

PubMed: 381

Cochrane Library: 128

Embase: 566

CINAHL: 159

WEB OF SCIENCE: 523

PubMed：

(
"Aquatic Therapy"[Mesh]
OR aquatic therap*[Title/Abstract]
OR aquatic exercise*[Title/Abstract]
OR water-based exercise*[Title/Abstract]
OR water exercise*[Title/Abstract]
OR hydrotherap*[Title/Abstract]
OR aquatic training[Title/Abstract]
OR aquatic rehabilitation[Title/Abstract]
OR water-based rehabilitation[Title/Abstract]
OR pool exercise*[Title/Abstract]
OR water aerobics[Title/Abstract]
OR water walking[Title/Abstract]
OR aqua jogging[Title/Abstract]
OR aquatic resistance exercise[Title/Abstract]
OR aquatic balance training[Title/Abstract]
OR swimming[Title/Abstract]
OR swim*[Title/Abstract]
)
AND
(
"Asthma"[Mesh]
OR asthma[Title/Abstract]
OR asthmatic*[Title/Abstract]
)

Cochrane Library

(aquatic therap* OR aquatic exercise* OR water-based exercise* OR water exercise* OR hydrotherap* OR aquatic training OR aquatic rehabilitation OR water-based rehabilitation OR pool exercise* OR water aerobics OR water walking OR aqua jogging OR aquatic resistance exercise OR aquatic balance training OR swimming OR swim*):ti,ab,kw AND (asthma OR asthmatic*):ti,ab,kw

Embase

(
'exp aquatic therapy'/exp
OR aquatic therap*:ti,ab,kw
OR aquatic exercise*:ti,ab,kw
OR water-based exercise*:ti,ab,kw
OR water exercise*:ti,ab,kw
OR hydrotherap*:ti,ab,kw
OR aquatic training:ti,ab,kw
OR aquatic rehabilitation:ti,ab,kw
OR water-based rehabilitation:ti,ab,kw
OR pool exercise*:ti,ab,kw
OR water aerobics:ti,ab,kw
OR water walking:ti,ab,kw
OR aqua jogging:ti,ab,kw
OR aquatic resistance exercise:ti,ab,kw
OR aquatic balance training:ti,ab,kw
OR swimming:ti,ab,kw
OR swim*:ti,ab,kw
)
AND
(
'exp asthma'/exp
OR asthma:ti,ab,kw
OR asthmatic*:ti,ab,kw
)

CINAHL

(
(MH "Aquatic Therapy+")
OR TI (aquatic therap* OR aquatic exercise* OR water-based exercise* OR water exercise* OR hydrotherap* OR aquatic training OR aquatic rehabilitation OR water-based rehabilitation OR pool exercise* OR water aerobics OR water walking OR aqua jogging OR aquatic resistance exercise OR aquatic balance training OR swimming OR swim*)
OR AB (aquatic therap* OR aquatic exercise* OR water-based exercise* OR water exercise* OR hydrotherap* OR aquatic training OR aquatic rehabilitation OR water-based rehabilitation OR pool exercise* OR water aerobics OR water walking OR aqua jogging OR aquatic resistance exercise OR aquatic balance training OR swimming OR swim*)
)
AND
(
(MH "Asthma+")
OR TI (asthma OR asthmatic*)
OR AB (asthma OR asthmatic*)
)

WEB OF SCIENCE

TS=(
"aquatic therap*"
OR "aquatic exercise*"
OR "water-based exercise*"
OR "water exercise*"
OR hydrotherap*
OR "aquatic training"
OR "aquatic rehabilitation"
OR "water-based rehabilitation"
OR "pool exercise*"
OR "water aerobics"
OR "water walking"
OR "aqua jogging"
OR "aquatic resistance exercise"
OR "aquatic balance training"
OR swimming
OR swim*
)
AND
TS=(
asthma
OR asthmatic*
)
